# Enlarged Mesenteric Gland and an Aneurism of the Anterior Mesenteric Artery

**Published:** 1892-07

**Authors:** J. E. Miller

**Affiliations:** Keyingham


### ENLARGED MESENTERIC GLAND AND AN ANEURISM OF THE ANTERIOR MESENTERIC ARTERY.

By J. E. Miller, M. R. C. V. S, Keyingham.

About eight o'clock on June 1st I was called upon to attend a filly (Olivette) which belonged to a well-known Yorkshire breeder, and had taken several prizes. On my arrival the animal presented the following symptoms: Countenance anxious, the head drooped, moved stiffly in hind quarters, respirations quickened, mucous membrane injected, ears cold, temperature 102, pulse 50, non-passage of fæces, unpleasant odor from mouth and serous discharge from the nostrils. On auscultation I found the heart and lung sounds much disturbed, and on percussion there was evidence of pain.

I gave stimulants and applied a liniment to side, thinking it was suffering from a violent cold, as it had been running out the night before. I called to see my patient in the evening; it seemed little or no better, so continued the treatment and gave a laxative. The following morning I found it dead. Being requested by the owner to make a post-mortem, I did so, and found the following: Skin and subcutaneous tissues were normal, but on opening the abdomen I found a dirty, watery fluid tinged with blood. On removing the large bowels and proceeding to the ileum, I found a large tumor with about a foot of the ileum attached to it, and the peritoneum here was much inflamed. Liver and kidneys were healthy, but the lungs were congested, the heart enlarged, and an ante mortem clot in it. The other parts of the body were comparatively healthy.

As I had never seen a tumor of this kind before (it weighing about 14 lbs.) my assistant forwarded it to Professor Walley, who sent the following character of it: That it was an enormously enlarged mesenteric gland, with a portion of the ileum and the anterior mesenteric artery attached to it, the latter being the subject of aneurism as the result of irritation by parasites (strongylus armatus), and the peritonitis was of septic or erysipelatous type. —*The Veterinary Record.*

